# Correlation Between End-Tidal Carbon Dioxide and Regional Cerebral Oxygen Saturation During Cardiopulmonary Resuscitation

**DOI:** 10.3390/jcm14113747

**Published:** 2025-05-27

**Authors:** Mateusz Putowski, Magdalena Dudzikowska, Wojciech Wieczorek, Michal Pruc, Lukasz Szarpak, Zbigniew Siudak

**Affiliations:** 1 Collegium Medicum, Jan Kochanowski University of Kielce, 25-369 Kielce, Poland; 2 Center for Innovative Medical Education, Jagiellonian University Medical College, 31-008 Cracow, Poland; 3 Department of Anesthesiology and Intensive Care, University Hospital, 31-501 Cracow, Poland; 4 Department of Emergency Medicine, Medical University of Warsaw, 02-005 Warsaw, Poland; 5 Department of Clinical Research and Development, LUXMED Group, 02-678 Warsaw, Polandlukasz.szarpak@kul.pl (L.S.); 6 Institute of Medical Science, Collegium Medicum, The John Paul II Catholic University of Lublin, 20-950 Lublin, Poland; 7 Henry JN Taub Department of Emergency Medicine, Baylor College of Medicine, Houston, TX 77030, USA

**Keywords:** in-hospital cardiac arrest (IHCA), near-infrared spectroscopy (NIRS), regional cerebral oxygen saturation (rSO_2_), end-tidal carbon dioxide (ETCO_2_), cardiopulmonary resuscitation (CPR)

## Abstract

**Background/Objectives:** Near-infrared spectroscopy (NIRS) enables the non-invasive assessment of cerebral oximetry, offering insights into the efficacy of oxygen supply to the brain. NIRS, when combined with other monitoring techniques such as capnography, may play a crucial role in advanced patient monitoring during sudden cardiac arrest and post-resuscitation treatment. This research assessed the relationship between end-tidal carbon dioxide (ETCO_2_) and regional cerebral oxygen saturation (rSO_2_) during cardiopulmonary resuscitation. **Methods:** The research was performed from 11 January 2023 until 31 January 2024, at the University Hospital in Poland. The cohort of responders included patients who had in-hospital cardiac arrest (IHCA). The Rapid Response Team attached the rSO_2_ and ETCO_2_ monitoring devices to each patient during cardiopulmonary resuscitation (CPR). The cohort included 104 patients. **Results:** The correlation coefficient between ETCO_2_ and rSO_2_ values was 0.641 (95% CI: 0.636–0.646), and during the last 4 min of CPR before ROSC, it was 0.873 (95% CI: 0.824–0.910). **Conclusions:** The positive correlation between ETCO_2_ and rSO_2_ may suggest that concurrent monitoring of both parameters during resuscitation might serve as a valuable predictor of CPR efficacy and the likelihood of achieving recovery of spontaneous circulation in a multimodal framework. In the lack of rapid ETCO_2_ monitoring capabilities, rSO_2_ may function as a simple and effective alternative for assessment.

## 1. Introduction

Cardiac arrest refers to the interruption of cardiac activity and the disruption of blood circulation. It may arise suddenly and can be linked to several conditions. If cardiac arrest persists for a prolonged period without the heart resuming activity, all essential functions cease. Consequently, it is crucial to restart the heart to sustain life. Cardiac arrest may transpire inside a healthcare institution, such as a hospital, or in an external environment. Cardiac arrest is a significant public health challenge globally. The yearly incidence of in-hospital cardiac arrest (IHCA) in the United States is estimated to be over 300,000 cases. This varies from 1.6 to 2.85 per 1000 hospital admissions, according to statistics from the United Kingdom and the United States, respectively [1]. The yearly incidence of out-of-hospital cardiac arrest (OHCA) in Europe ranges from 67 to 170 per 100,000 population, and over 350,000 incidents occur annually in the United States [2,3]. Prioritizing high-quality chest compressions, defibrillation, and the identification and treatment of reversible causes is essential in managing both IHCA and OHCA. A key factor of effective cardiopulmonary resuscitation (CPR) is the assessment of end-tidal CO_2_ (ETCO_2_) [4]. Carbon dioxide is produced in tissues as a result of aerobic metabolism, diffusing from cells into the blood, is transported via the venous route to the lungs, and then is removed by ventilation. The factors that determine the ETCO_2_ measurement results are CO_2_ production, pulmonary perfusion, alveolar ventilation, and cardiac output. Its accurate value is 35–45 mmHg [5]. According to current knowledge, capnography provides information on the effectiveness of chest compressions due to the positive correlation of ETCO_2_ with cardiac index, coronary perfusion pressure, and cerebral blood flow [6,7]. However, the value of ETCO_2_ during resuscitation may be affected by various factors, such as the supply of adrenaline or other drugs from the group of vasopressors, which can lead to a temporary decrease in the value of ETCO_2_, whereas the administration of sodium bicarbonate results in a marked increase in this parameter. This parameter is also markedly reduced during CPR in the presence of pulmonary embolism, and an ETCO_2_ over 10 mmHg during sudden cardiac arrest (SCA) enhances the likelihood of return of spontaneous circulation (ROSC) and survival, but its utility has not yet been fully confirmed. Therefore, ETCO_2_ should be considered as an element of the multimodal approach in CPR monitoring [4,8,9,10]. It is also important to recognize that to accurately evaluate this parameter, the airways must be adequately cleared, potentially causing a considerable delay in its measurement during the early phase of CPR.

Near-infrared spectroscopy (NIRS) is used to assess the efficacy of microcirculation inside a specific organ, including principles of quantum mechanics and nonlinear optics. The measurement involves positioning the sensor on the patient’s forehead using a specialized electrode. Regional cerebral saturation (rSO_2_) ranges from 70% to 80% venous blood saturation. Oxygen saturation of venous blood is an indicator reflecting the balance between the demand and use of oxygen in tissues. Consequently, NIRS is used to evaluate organ perfusion and tissue oxygen consumption. Research validates its efficacy in tracking the progression of CPR; nonetheless, the findings remain unclear [11,12].

This research aimed to evaluate the association between ETCO_2_ and rSO2 during cardiopulmonary resuscitation. Our results may facilitate the correlation between these parameters, thereby enhancing the efficiency of CPR quality monitoring and the prediction of ROSC. Furthermore, we aim to rectify the inadequacy in the multimodal approach. The simple installation of the rSO_2_ sensor enables prompt monitoring from the beginning of CPR. This study is the only known assessment in which both measurements were simultaneously collected from a single monitoring device.

## 2. Materials and Methods

The research was conducted from 11 January 2023 to 31 January 2024, at the University Hospital in Krakow, Poland. The cohort of responders included individuals who had sudden cardiac arrest while hospitalized. The Ethics Committee of Kielce, Poland, authorized the research (number 11/2023). The Rapid Response Team (RRT) established a connection between the rSO_2_ and end-tidal carbon dioxide concentration monitoring devices for each patient within 5 min of diagnosing in-hospital cardiac arrest.

### 2.1. Inclusion and Exclusion Criteria

Inclusion criteria included sudden cardiac arrest in a hospitalized patient, age over 18 years, and patients in normothermia.

Exclusion criteria included no connection of the rSO_2_ and ETCO_2_ sensor or malfunction (electrode or device) during CPR, patients admitted to the hospital during CPR, cardiac arrest occurring in hospitalized patients in the ICU, cardiac arrest occurring in the operating theater, placement of the rSO_2_ monitoring sensor more than 5 min after the occurrence of cardiac arrest, patients suspected of internal or external hemorrhage, and information regarding palliative care.

### 2.2. Cardiopulmonary Resuscitation

The CPR procedure was performed in accordance with the 2021 Guidelines of the European Resuscitation Council. After receiving the report and reaching the call site, where the patient’s IHCA took place, the RRT took over the management of CPR from the nursing and medical staff of the department. The RRT comprises a specialist in anesthesiology and critical care, a physician in training in anesthesiology and intensive care, and two paramedics. Throughout the research, all team members had valid certification in Advanced Resuscitation Procedures (ALS). The electrodes for rSO_2_ monitoring were positioned following the manufacturer’s guidelines by a selected trained team member, about 1 cm above the patient’s eyebrows on the forehead. The ETCO_2_ was ultimately monitored by a sensor positioned between the bacteriostatic filter of the intubation tube and the self-inflating bag or ventilator. The rSO_2_ and ETCO_2_ measurements were recorded at 2 sec intervals using the Masimo Open Connect MOC-9 Module. Ventilation was performed asynchronously via a self-inflating bag or a CPR ventilator at a rate of 10 breaths per minute. The whole process of in-hospital cardiopulmonary resuscitation has been recorded in accordance with the Utstein Resuscitation Registry Template for the IHCA protocol [13]. The mean time from cardiac arrest recognition to initiation of basic life support (BLS) by ward staff was estimated at under 1 min. An automated external defibrillator (AED) was available in all monitored units, and defibrillation was performed when indicated prior to RRT arrival. The Rapid Response Team (RRT) arrived at the scene with a median response time of 4.0 min (IQR: 3.0–4.9 min). Advanced life support was initiated immediately upon arrival. Endotracheal intubation was performed without delay, and mechanical ventilation or self-inflating bag ventilation was provided asynchronously at 10 breaths per minute with 100% FiO_2_. The ETCO_2_ sensor was placed within 4 min after RRT arrival, and rSO_2_ electrodes were applied simultaneously. All procedures strictly followed the 2021 ERC Guidelines for in-hospital cardiac arrest management.

### 2.3. Statistical Analysis

We employed the Shapiro–Wilk test to evaluate the distribution of the gathered data for each variable (ETCO_2_ and rSO_2_) in both the ROSC and non-ROSC groups. Furthermore, histogram plots were examined to visually assess the distribution’s form. Both the statistical tests and visual assessments demonstrated that the data for rSO_2_ and ETCO_2_ in both groups conformed to a normal distribution, thereby justifying the application of parametric methods for subsequent analysis.

We employed the Student’s *t*-test to compare the two independent groups (ROSC vs. non-ROSC) based on the distribution assessment. A generic linear model for repeated measures was utilized to evaluate temporal variations in measurements during CPR. A post hoc analysis employing Bonferroni correction was performed to evaluate pairwise comparisons among designated time points: M4 (mean value 4 min prior to ROSC), M3, M2, and M1 (1 min prior to ROSC).

We evaluated the correlations between ETCO_2_ and rSO_2_ using the Pearson linear correlation coefficient, classifying them as follows: 0–0.19 = destitute; 0.20–0.39 = poor; 0.40–0.59 = moderate; 0.60–0.79 = strong; 0.80–1.0 = forceful correlation.

Missing data (≤10% per patient) were addressed by linear interpolation, utilizing the mean of the neighboring values preceding and succeeding the gap, based on the assumption of missing at random (MAR).

Descriptive statistics were presented as means (standard deviation, SD), medians (interquartile range, IQR), and 95% confidence intervals (CIs) when applicable. A *p*-value less than 0.05 was deemed statistically significant. All statistical analyses were conducted utilizing IBM SPSS Statistics, version 29.0 for macOS.

### 2.4. Confounding Variables

The supply of sodium bicarbonate (NaHCO_3_) was considered a factor that could have a significant impact on the ETCO_2_ result [5,14]. A period of 7 min after drug administration was excluded from the analyses. The drug was administered to 21 patients.

## 3. Results

The total duration of the analyzed signals was 1776 min. The mean value of rSO2 (SD) during CPR for patients who achieved ROSC was 63.8% (7.4), and for patients who did not achieve ROSC, it was 35.6% (5.6) (*p* < 0.001). The mean (SD) ETCO2 value in the ROSC group compared to the non-ROSC group was 26 mmHg (4.9) vs. 17 mmHg (4.8) (*p* < 0.001). The characteristics of the patients are presented in Table 1. A statistically significant strong positive correlation was observed between ETCO2 and rSO2 values (*p* < 0.001). The correlation coefficient was 0.641 (95% CI: 0.636–0.646). When the ETCO2 value rises, the rSO2 value also increases. After eliminating data after NaHCO_3_ administration (*n* = 21), the correlation coefficient increased to 0.648 (95% CI: 0.643–0.652, *p* < 0.001). For the group of patients without ROSC, the correlation coefficient was 0.317 (95% CI: 0.307–0.327; *p* < 0.001), indicating a slight positive correlation. After excluding data after NaHCO_3_ administration (*n* = 6), the correlation coefficient increased to 0.319 (95% CI: 0.309–0.329) (*p* < 0.001). In the ROSC group, the correlation coefficient was 0.228 (95% CI: 0.215–0.240) (*p* < 0.001), indicating a slight positive correlation. After excluding the growth phases for ETCO2 resulting from the administration of NaHCO3 (*n* = 15), the correlation increased to 0.253 (95% CI 0.241–0.266) (*p* < 0.001). The distribution of initial rhythms between the ROSC and non-ROSC groups revealed a higher proportion of shockable rhythms (VF or pVT) in the ROSC group compared to the non-ROSC group (20% vs. 12%, respectively). Conversely, non-shockable rhythms (asystole and PEA) were predominant in both groups but significantly more common in the non-ROSC group (88%) than in the ROSC group (80%).

### Correlation Between rSO2 and ETCO2 Prior to ROSC

**Figure 1:**
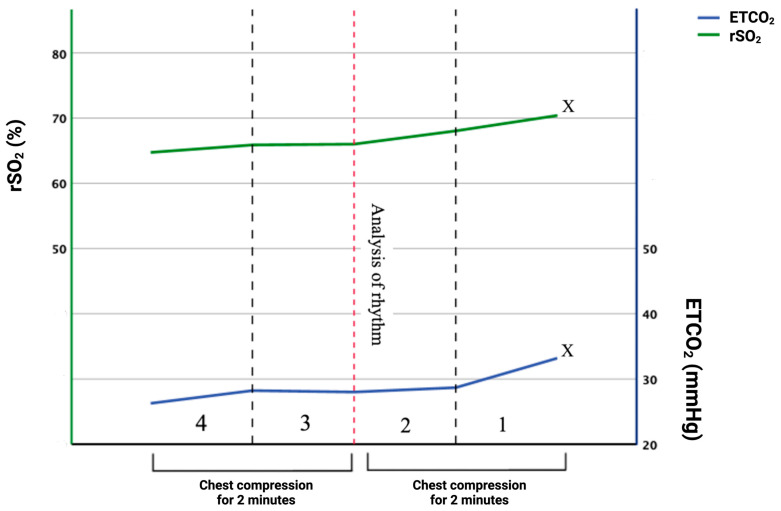
Mean values of rSO_2_ and ETCO_2_ in the ROSC group (*n* = 54) from the last 4 min before the end of CPR. The moment of rhythm assessment is marked with a red dashed line. X means the moment of interruption of CPR and the finding of ROSC.

The last 4 min before the ROSC in the patient group were evaluated to determine whether a rapid elevation in both measures might indicate ROSC. The average values of rSO_2_ and ETCO_2_ were assessed over four intervals. The values are designated as M4, M3, M2, and M1. Statistically significant variations were observed between consecutive rSO_2_ and ETCO_2_ observations. No variation in ETCO_2_ was seen between the mean values at the 2nd and 3rd minutes of resuscitation before the onset of ROSC. The data indicate that both rSO_2_ and ETCO_2_ levels increased as they neared the ROSC finding. The rSO_2_ value between M4 and M1 (64.6% vs. 70.4%) exhibited a variation of 5.8 percentage points, representing a 9% increase. The ETCO_2_ measurement between M4 and M1 (26.2 mmHg vs. 32.2 mmHg) showed a variation of 6 mmHg, corresponding to a change of 23%. ETCO_2_ exhibited a more significant variation than rSO_2_ in the last 4 min before the achievement of ROSC. The correlation coefficient of ETCO_2_ to rSO_2_ in patients who achieved ROSC in the last 4 min of CPR was 0.873 (95% CI: 0.824–0.910), indicating a robust correlation and demonstrating that both parameters rise as the return of circulation, confirmed by the pulse in the central arteries, is approached (Table 2, Figure 1).

In contrast to the ROSC group, the non-ROSC group exhibited only minimal increases in rSO_2_ and ETCO_2_ values across the M4 to M1 time points, with a weak correlation (r = 0.317; 95% CI: 0.307–0.327). These findings are summarized in Table 3 and reinforce the hypothesis that a dynamic rise in both rSO_2_ and ETCO_2_ is associated with the successful return of spontaneous circulation.

## 4. Discussion

The results underscore that the correlation of rSO_2_ with ETCO_2_ during resuscitation may serve as an essential tool for enhancing the resuscitation process, monitoring patient condition, and increasing the chance of successful resuscitation and favorable neurological outcomes. In the context of IHCA, when CPR is performed by a nurse–physician team, the use of NIRS monitoring enables the tracking of rSO_2_ values from the first minutes of CPR, prior to the availability of ETCO_2_ measurements [15]. Monitoring physiological signals during CPR may improve the assessment of resuscitation quality and help predict ROSC and long-term neurological outcomes. Although comparisons with out-of-hospital cardiac arrest (OHCA) research provide useful insights into the general physiology of resuscitation, it is crucial to interpret these data cautiously. IHCA and OHCA markedly differ regarding response time, initial rhythm prevalence, pre-existing comorbidities, and monitoring capacity. Consequently, the extension of OHCA findings to the IHCA group in our investigation is intended just to furnish physiological context, rather than to establish direct clinical equivalency.

Capnography, although a non-invasive technique to measure ETCO_2_ during CPR, necessitates an invasive procedure such as endotracheal intubation or using the epiglottic approach. A recent secondary analysis of a large OHCA trial indicated that ETCO_2_ trends over time provide prognostic information. ETCO_2_ values were significantly higher in patients who achieved ROSC compared to those who did not, as resuscitation progressed, and an increasing ETCO_2_ slope was independently linked to ROSC [16]. These data confirm that capnography offers immediate feedback on systemic perfusion and can direct resuscitative measures. Nonetheless, ETCO_2_ possesses limitations as a predictive measure. Its measurements may be distorted by alterations in ventilation (rate and tidal volume), airway condition, pulmonary disease, or metabolic influences [7,17]. Excessive ventilation may reduce ETCO_2_ levels despite sufficient perfusion, while CO_2_ accumulation during extended downtimes or bicarbonate administration might temporarily increase ETCO_2_ irrespective of circulation. Consequently, although ETCO_2_ is essential for monitoring CPR, it offers restricted prognostic accuracy when utilized alone [17]. Moreover, the sensor may often get contaminated with respiratory secretions, perhaps resulting in inaccurate readings or temporary failures. Extreme values or distinct trends (consistent increase or abrupt decrease) provide more insights than any singular absolute measurement, and additional research is required to develop reliable baselines for clinical decision-making. Experimental studies have shown that during CPR, ETCO2 correlates well with the Cardiac Index (0.79; *p* < 0.001) [16] with coronary perfusion pressure (0.78; *p* < 0.001) [16] and blood flow through the brain (0.64; *p*  = 0.01) [7]. The published document of the American Heart Association recommends monitoring ETCO_2_ as the basic indicator during CPR and suggests that a target ETCO_2_ > 20 mmHg should be pursued [18].

NIRS is commonly used to optimize general anesthesia during open-heart surgery [19,20] for patients with head traumas [21] and during carotid endarterectomy [22]. The efficacy of NIRS has been validated in clinical scenarios with increased risk of cerebral ischemia, and in recent years, rSO_2_ has been used to monitor venous–arterial and venous–venous extracorporeal membrane oxygenation [23]. Numerous observational studies indicate that the mean rSO_2_ in patients who achieve ROSC is significantly elevated compared to those who do not [18,19]. In the study by Parnia et al., the average rSO_2_ during CPR was approximately 52% in patients who achieved ROSC, compared to around 41% in those who did not. Moreover, Parnia et al. indicated that patients exhibiting favorable neurological status at discharge (Cerebral Performance Category 1–2) demonstrated a significantly elevated mean rSO_2_ during CPR (~56%) in contrast to those with adverse outcomes (~44%). Additionally, the proportion of CPR duration exceeding a crucial rSO_2_ threshold is also indicative: In that study, sustaining rSO_2_ > 50% for ≥60% of the resuscitation correlated with a 98% negative predictive value for unfavorable outcomes [20]. These data indicate that sufficient cerebral perfusion during CPR correlates with less neurological damage. Conversely, ETCO_2_ has not been demonstrated to forecast neurological outcomes directly beyond its function in attaining ROSC, as the restoration of circulation is a precondition for brain recovery. It is essential to recognize that post-resuscitation treatment and additional factors eventually influence neurological outcomes. A review of 26 studies indicated that the pooled mean rSO_2_ in patients with ROSC was approximately 41%, in contrast to about 30% in those without ROSC (*p* = 0.009) [12]. Importantly, cerebral oximetry trends appear to carry prognostic value. Patients who achieve ROSC exhibit a more significant increase in rSO_2_ during continuous CPR compared to those who do not. In a substantial cohort of OHCA cases, the median increase in rSO_2_ was 17% in cases with a ROSC, compared to 8% in other cases. An increase greater than 15% from baseline was associated with an odds ratio of approximately 4.9 for predicting ROSC [20]. A sudden increase in rSO_2_, such as from the 20–30% range to over 50% within a few minutes, may indicate ROSC, similar to the spike in ETCO_2_, and can be identified without the necessity of an arterial pulse or advanced airway [20,21]. The findings align with these trends: Successful resuscitation cases exhibited elevations in rSO_2_ toward the conclusion of the process, while consistently low rSO_2_ indicated a failure in resuscitation efforts. Our data, along with previous studies, indicate that rSO_2_ may serve as a more sensitive early marker of perfusion return compared to ETCO_2_.

In a study conducted in an emergency department on cardiac arrest, cerebral oximetry demonstrated superior performance compared to ETCO_2_ in predicting ROSC, with an area under the curve of 0.89 versus 0.77 in the final minute of CPR [22]. Our IHCA cohort exhibited enhanced predictive performance of rSO_2_ compared to ETCO_2_, evidenced by a significantly higher area under the curve for ROSC. The findings suggest that cerebral saturation serves as a direct measure of end-organ (brain) perfusion, which is influenced by cardiac output and arterial oxygen content. If CPR produces any forward flow, rSO_2_ will increase, assuming that the oxygen delivered is adequate, thereby reflecting the sufficiency of both circulation and oxygenation [23,24]. ETCO_2_ indicates the delivery of CO_2_ to the lungs. Even with improved perfusion, it may not rise if ventilation is poor or metabolic CO_2_ production is low. Combining both metrics provides a more comprehensive understanding: In our study, every patient who achieved ROSC exhibited improvements in both ETCO_2_ and rSO_2_, whereas those without ROSC demonstrated corresponding declines. The result suggests a strong relationship between systemic and cerebral perfusion during CPR, consistent with findings from experimental models that demonstrate an increasing correlation between ETCO_2_ and rSO_2_ values over the course of resuscitation, nearing r ≈ 1.0 in the later stages of CPR [25]. NIRS, when combined with other monitoring techniques like capnography, may play a crucial role in the advanced monitoring of critically ill patients and those experiencing SCA, offering insights into hemodynamic state and cerebral perfusion [11]. Moreover, the simplicity of sensor installation might be significantly crucial when ETCO_2_ measurement is not easily accessible.

There is a very limited number of studies that determine the correlation between ETCO_2_ and rSO_2_. This may be due to the limited availability of devices that have the ability to measure both of these parameters at the same time. The device used in our research has two modules, enabling the simultaneous monitoring of these two indicators and the collection of data from the same moment of measurement. We observed a strong correlation between rSO_2_ and ETCO_2_ during CPR, suggesting that both metrics reflect the underlying hemodynamic quality. Research using a porcine model established a significant correlation between ETCO_2_ and cardiac output during resuscitation, with a value of 0.83 (*p* < 0.001) (95% CI: 0.67–0.92), and a positive correlation between rSO2 and cardiac output during resuscitation, with a value of 0.50 (*p* = 0.004) (95% PU: 0.16–0.73). In this study, ETCO_2_ demonstrated a more robust association than rSO_2_. Researchers underscored the need to monitor both values in a hemodynamically unstable patient for the prompt detection of cardiac arrest, hence facilitating the early commencement of life-saving interventions [26]. Moreover, there are pivotal situations in which one monitor may identify problems that the other fails to recognize. For instance, if an endotracheal tube is dislodged or the patient experiences hyperventilation, ETCO_2_ will sharply decrease despite the continuity of chest compressions (and consequently cerebral perfusion); rSO_2_ could inform clinicians that brain oxygenation is still sufficient or is declining gradually during that period. Conversely, an elevation in ETCO_2_ without a corresponding increase in rSO_2_ may raise concerns regarding inadequate oxygen delivery or cerebral perfusion; for example, CO_2_ may be circulating from peripheral tissues, but the brain is not receiving sufficient blood flow or oxygen content. Using both methods together helps confirm that brain and systemic blood flow are adequate. Resuscitation experts have proposed a combination technique as part of “physiology-guided” CPR, in which ETCO_2_, arterial pressure, and, if accessible, cerebral oximetry are adjusted in real time to enhance perfusion [7]. Significantly, in contrast to capnography, NIRS monitoring does not necessitate the cessation of chest compressions or the establishment of an advanced airway, allowing for its implementation from the initiation of resuscitation, including during bag-mask breathing [5,21]. The strategy may be especially beneficial in hospital environments where NIRS devices are more readily available (such as operating rooms and ICUs) and where qualified workers can analyze various data streams. By monitoring both ETCO_2_ and rSO_2_, the team can more reliably detect ROSC, as a simultaneous increase in both metrics serves as a robust signal of ROSC, hence minimizing premature interruptions for pulse assessments. In our IHCA instances, we saw that dual monitoring offered confidence when both levels improved concurrently and indicated possible issues when they varied. It is important to acknowledge that while the qualitative integration of ETCO_2_ and rSO_2_ enhances monitoring, the additional prognostic significance of their combined use remains under investigation. In a porcine arrest model, the integration of rSO_2_ and ETCO_2_ did not markedly improve predictive accuracy for favorable neurologic outcomes compared to rSO_2_ or ETCO_2_ individually [25], mostly because both metrics provided analogous information at the conclusion of CPR. Similarly, in clinical investigations, the measure that first attains an extreme threshold (either extremely low or very high) frequently prompts clinical actions, complicating the demonstration that the second parameter influences outcomes. Nonetheless, a multimodal approach is advantageous for personalized care, as it enables responders to identify and rectify certain deficiencies in CPR. For instance, if ETCO_2_ is diminished despite satisfactory rSO_2_, clinicians may prioritize optimizing ventilation or assessing the airway; conversely, if rSO_2_ is reduced despite sufficient ETCO_2_, attention may be directed towards enhancing cerebral perfusion (e.g., improved chest recoil, vasopressor administration, or head positioning to augment cerebral blood flow). This personalized approach shows the future of resuscitation: moving from a standard protocol to goal-directed CPR using real-time data [7].

The current AHA and European guidelines do not endorse cerebral oximetry for regular neuroprognostication due to inadequate data [23]. Nevertheless, near-infrared monitoring continues to serve as a research instrument for the early detection of brain perfusion, and exceedingly low rSO_2_ values during CPR (e.g., consistently < 20–25%) may assist in identifying patients with a significant probability of severe neurological impairment or futility [19]. Future procedures may integrate rSO_2_ with existing post-arrest prognostic evaluations, including neurological examinations and EEG, in a multimodal approach.

The positive correlation we have shown indicates that both of these parameters are congruent with one another. However, ETCO_2_ can be affected by many confounding variables [5]. We have shown that in the group of patients achieving ROSC, both these parameters maintain elevated levels throughout the resuscitation period, in contrast to the group without ROSC. In the research conducted by Kämäräinen et al., cerebral saturation was assessed alongside resuscitation metrics, including the depth and frequency of chest compressions and ventilation rate. It was observed that the rSO_2_ value remained diminished, even during high-quality CPR [27,28,29]. Our study also verifies that rSO2 levels are decreased in patients who have not attained ROSC and elevated throughout the CPR period in the ROSC group. The assessment of rSO_2_ seems to be a more stable measure than ETCO_2_ and is unresponsive to pharmacological interventions or ventilation; nonetheless, more study is required [30]. A crucial result is that a substantial simultaneous rise in both measures may serve as a predictor of ROSC during the last period of CPR and might significantly enhance the multimodal monitoring method.

### Limitations

This research offers significant insights; nonetheless, it is crucial to recognize numerous limitations. The restricted sample size and single-center nature of this research are significant limitations that could limit the generalizability of the findings and diminish the statistical power to detect deeper differences. Furthermore, the research assumed that CPR performed by the RRT was carried out with adequate chest compressions and ventilation. Occasionally, there may have been unrecorded temporary disruptions in chest compressions or ventilation caused by intubation or other resuscitative interventions during ALS. NIRS mainly assesses the superficial layers of the brain, and when comparing studies with other researchers, it is essential to identify the kind of equipment used, since different models may provide disparate findings. The intervals of adrenaline administration, which may influence ETCO_2_ levels without impacting rSO2, were also not taken into account. Another restriction is the exclusive focus on ROSC, neglecting neurological prognosis and long-term prognostic evaluation. It is crucial to highlight that both rSO_2_ and ETCO_2_ are surrogate indicators influenced by various interrelated and independent physiological processes. These encompass cardiac output, pulmonary blood flow, alveolar-capillary gas exchange, CO_2_ generation at the tissue level, and regional cerebral perfusion dynamics. All of these measures are affected by breathing techniques, metabolic condition, vasopressor administration, and underlying pathology. While we concentrated on the direct relationship between rSO_2_ and ETCO_2_, a more extensive multiparametric model that includes simultaneous measurements—such as cardiac output (e.g., through echocardiography or invasive pressure monitoring), arterial oxygen saturation (SpO_2_), FiO_2_, lactate concentrations, or cerebral perfusion indices—might provide enhanced predictive capability for ROSC and neurological outcomes. Future research should incorporate these characteristics to enhance real-time prognostication and improve physiology-guided CPR techniques.

## 5. Conclusions

The strong correlation between ETCO_2_ and rSO_2_ may suggest that simultaneous monitoring of both parameters during resuscitation might serve as a valuable predictor of CPR efficacy and the likelihood of achieving ROSC in a multimodal approach. In the lack of rapid ETCO_2_ monitoring capabilities, rSO_2_ may function as a simple and effective alternative for monitoring.

## Figures and Tables

**Table 1:** Characteristics of patients divided into study groups (non-ROSC, ROSC).

Variables		IHCA (*n* = 104)	
		Non-ROSC (*n* = 50)	ROSC (*n* = 54)
Sex	Male (%)	34 (68%)	32 (59%)
Age (years)	Me (IQR)	69 (62; 75)	70 (63; 75)
	<65 (years)	17 (34%)	15 (28%)
	≥65 (years)	33 (66%)	39 (72%)
Duration of resuscitation (min)	Me (IQR)	25 (21; 30)	15 (10; 20)
	Min	15	5
	Max	40	31
rSO_2_ (%)	M (SD)	35.6 (5.6)	63.8 (7.4)
	Min	19	38
	Max	56	84
ETCO_2_ (mmHg)	M (SD)	17 (4.8)	26 (4.9)
	Min	4	11
	Max	40	64
Initial rhythm	Asystole (%)	20 (40%)	13 (24%)
	PEA (%)	24 (48%)	30 (56%)
	VF (%)	5 (10%)	7 (13%)
	pVT (%)	1 (2%)	4 (7%)

Legend: ROSC—return of spontaneous circulation; n—group size; SD—standard deviation; M—mean; Me—median; Min—minimum value; Max—maximum value; IQR—25 quartile, 75 quartile; rSO2—regional cerebral oxygen saturation; ETCO_2_—end-tidal carbon dioxide; PEA—pulseless electrical activity; pVT—pulseless ventricular tachycardia; VF—ventricular fibrillation.

**Table 2:** Average values of rSO_2_ and ETCO_2_ from the last 4 min of resuscitation.

ROSC *n* = 54					
Variable	M4 (SD)	M3 (SD)	M2 (SD)	M1 (SD)	*p* for Post Hoc Test
rSO_2_ (%)	64.6 (6.9)	65.8 (6.4)	68 (6.3)	70.4 (5.6)	M4–M3 *p* < 0.001 M3–M2 *p* < 0.001 M2–M1 *p* = 0.003
ETCO_2_ (mmHg)	26.2 (4.6)	28.2 (5.4)	28.8 (5.8)	32.2 (5.1)	M4–M3 *p* < 0.001 M3–M2 *p* = 1.000 M2–M1 *p* < 0.001

Legend: rSO_2_—regional cerebral oxygen saturation; ETCO_2_—end-tidal carbon dioxide; ROSC—return of spontaneous circulation; SD—standard deviation; M—mean; M4—indicates the average from 4th minute before the occurrence of ROSC; M3—indicates the average from 3rd minute before the occurrence of ROSC; M2—indicates the average from the 2nd minute before the occurrence of ROSC; M1—indicates the average from the last minute before the occurrence of ROSC.

**Table 3:** Mean rSO_2_ and ETCO_2_ values for ROSC and non-ROSC groups over the last 4 min of CPR.

Group	Parameter	M4 (Mean ± SD)	M3	M2	M1	ΔM4–M1	Pearson r
ROSC (*n* = 54)	rSO_2_ (%)	64.6 ± 6.9	65.8	68.0	70.4	+5.8	0.873
	ETCO_2_ (mmHg)	26.2 ± 4.6	28.2	28.8	32.2	+6.0	0.873
non-ROSC (*n* = 50)	rSO_2_ (%)	34.7 ± 5.2	34.9	35.2	35.3	+0.6	0.317
	ETCO_2_ (mmHg)	16.3 ± 4.1	16.5	16.6	16.8	+0.5	0.317

Legend: ΔM4–M1—change from 4th minute to 1st minute before ROSC assessment; Pearson r—correlation coefficient for M4–M1 in each group.

## Data Availability

The data that support the findings of this study are available from the corresponding author upon reasonable request.

## Abbreviations

The following abbreviations are used in this manuscript:

AHA	American Heart Association
ALS	Advanced life support
CI	Confidence interval
CPR	Cardiopulmonary resuscitation
EEG	Electroencephalogram
ETCO_2_	End-tidal carbon dioxide
IHCA	In-hospital cardiac arrest
IQR	Interquartile range
MOC-9	Masimo Open Connect MOC-9 Module
NaHCO_3_	Sodium bicarbonate
NIRS	Near-infrared spectroscopy
OHCA	Out-of-hospital cardiac arrest
PEA	Pulseless electrical activity
pVT	Pulseless ventricular tachycardia
ROSC	Return of spontaneous circulation
rSO_2_	Regional cerebral oxygen saturation
RRT	Rapid Response Team
SD	Standard deviation
VF	Ventricular fibrillation
